# Sensitive HPV detection in oropharyngeal cancers

**DOI:** 10.1186/1471-2407-9-440

**Published:** 2009-12-15

**Authors:** David M Winder, Siolian LR Ball, Katie Vaughan, Nashat Hanna, Yin Ling Woo, Jürgen-Theodor Fränzer, Jane C Sterling, Margaret A Stanley, Holger Sudhoff, Peter KC Goon

**Affiliations:** 1 Dept of Pathology, University of Cambridge, Tennis Court Road, Cambridge CB2 1QP, UK; 2 Dept of GU/HIV Medicine, St. Mary's Hospital, Praed Street, London W2 1NY, UK; 3 Bielefeld Academic Teaching Hospital, Department of Otorhinolaryngology, Teutoburgerstraße 50, D-33604 Bielefeld, Germany

## Abstract

**Background:**

Human papillomaviruses (HPV) are the aetiological agents of certain benign and malignant tumours of skin and mucosae; the most important of which is cervical cancer. Also, the incidence of ano-genital warts, HPV-anal cancer and oropharyngeal cancers are rising. To help ascertain a useful PCR detection protocol for oropharyngeal cancers, we directly compared three commonly used primer sets in detection of HPV from different clinical samples.

**Methods:**

We compared PGMY09/11, MY09/11 and GP5+/6+ primers sets in PCRs of 34 clinically diagnosed samples of genital warts, cervical brushings (with associated histological diagnosis) and vulval biopsies. All negative samples were subsequently tested using the previously reported PGMY/GP PCR method and amplicons directly sequenced for confirmation and typing. An optimised PCR protocol was then compared to a line blot assay for detection of HPV in 15 oropharyngeal cancer samples.

**Results:**

PGMY09/11 primers detected HPV presence in more cervical brushing (100%) and genital wart (92.9%) samples compared to MY09/11 (90% and 64.3%) and GP5+/6+ (80% and 64.3%) primer sets, respectively. From vulval biopsies, HPV detection rates were: MY09/11 (63.6%), GP5+/6+ (54.5%) and PGMY09/11 (54.5%). PGMY/GP nested PCR demonstrated that HPV was present, and direct sequencing confirmed genotypes. This nested PCR protocol showed detection of HPV in 10/15 (66.7%) of oropharyngeal cancer samples.

**Conclusions:**

PGMY09/11 primers are the preferred primer set among these three for primary PCR screening with different clinical samples. MY09/11 and GP5+/6+ may be used (particularly for cervical samples) but demonstrate lower detection rates. A nested PCR approach (i.e. a PGMY-GP system) may be required to confirm negativity or to detect low levels of HPV, undetectable using current primary PCR methods, as demonstrated using oropharyngeal cancer samples.

## Background

Strong epidemiological and molecular evidence has demonstrated human papillomavirus (HPV) to be the aetiological agent of both benign (warts, papillomas) and malignant tumours (subsets of ano-genital and oropharyngeal carcinomas) [1]. It has been estimated that HPV accounts for over 5% of total annual worldwide cancers [2]. The persistence of high-oncogenic risk subtypes has been demonstrated to be a necessary but not sufficient cause of cervical cancer [3]; the principal cancer of women in the developing world and second commonest female cancer worldwide [4,5] (~510,000 cases with 288,000 deaths annually) [6].

More recently, however, strong evidence linking HPV to the development of approximately 20-50% (depending on the anatomical site) [7,8] of head and neck cancers has been accumulating [9-11]. This is an issue of great global importance as head and neck cancer is the 5^th ^most common cancer in the world [12,13]. Mortality has not improved substantially over the last few decades [14], due to late diagnosis (75% of cases) and/or recurrent primary malignancies, and remains at 40-50% at 5 years [15,16].

HPV subtypes are frequently classified as high-risk (HR-HPV) or low-risk (LR-HPV) for the development of cervical cancer [5,17]. The recent development and introduction of prophylactic HPV vaccines for cervical cancer has now provided fresh impetus to the detection of HPV infection and associated disease in the community, as government initiatives to track the efficacy of vaccination programmes commence. There is a strengthening case for the general introduction of the vaccines into the community for prevention of HPV-associated non-cervical cancers [18].

Currently, the most common tests used to detect genital HPV in cervical samples are the hybridization assay Hybrid Capture II (HCII, Digene HPV test, QIAGEN Ltd, UK), and PCR systems with the degenerate primer sets MY09/11 and GP5+/6+ [19-22]. These target the conserved HPV L1 region and detect a broad range of subtypes, subsequent sequencing allowing specific subtype identification. However, in multiple HPV infections, sequencing may miss less prevalent subtypes [23]. The advent of the PGMY09/11 primer set allowed detection of increased sensitivity and a broader range of HPV types, especially in multiply infected cervico-lavage samples [24]. Furthermore, nested PCR with PGMY/GP+ primer sets was tested in cervical samples and, compared to MY/GP+, found to provide a wider range of detectability, greater sensitivity and performed better in characterisation of multiple infections [25]. Large meta-analyses have failed to identify an optimal PCR system for HPV detection on oropharyngeal cancers [7,8].

In this study, we sought to ascertain an efficient protocol for HPV detection in different types of clinical samples. We evaluated HPV detection in DNA from three common types of genital clinical samples; directly comparing genital warts, CIN (cervical intra-epithelial neoplasia) brushings and VIN (vulval intra-epithelial neoplasia) lesions using the MY09/11, GP5+/6+ and PGMY09/11 primer sets in primary PCR and with subsequent comparison with a nested PCR approach (PGMY-GP) [25]. We then compared detection of HPV from oropharyngeal cancers with a commonly used and commercially available line blot assay (Linear Array™, Roche Diagnostics Ltd, UK) which discerns 37 different HPV genotypes. The significance of our findings on sensitive PCR detection of HPV from this range of clinical samples, and in particular, oropharyngeal cancers, is discussed.

## Methods

### Study samples

Clinical samples were obtained from patients attending the Departments of Gynaecological Oncology, Dermatology, Addenbrooke's Hospital, Cambridge, UK, Department of GU/HIV Medicine, St Mary's Hospital, London, UK and the Bielefeld Academic Teaching Hospital, Department of Otorhinolaryngology, Bielefeld, Germany. All patients gave written informed consent and ethical approval was obtained from local research ethics committees. All experiments were performed in the Department of Pathology at the University of Cambridge.

Genital warts were excised as part of treatment, and snap-frozen until DNA extraction. A Cervex™ brush (Rovers Medical Devices B.V. Oss, The Netherlands) was used for cervical sampling, agitated in PreservCyt™ solution (Hologic UK Ltd.) for fixation and preservation of cells and stored at 4°C until DNA extraction. A concomitant biopsy was taken for CIN staging. All samples were processed blind. VIN was diagnosed histopathologically from vulval biopsies, with some tissue processed for DNA. Oropharyngeal cancer samples, acquired from Germany, were snap-frozen in liquid nitrogen and transported to the UK on dry ice prior to DNA extraction. All samples were diagnosed by the resident consultant histopathologist of the institutions involved.

### DNA extraction from samples

#### Clinical Wart Samples

Total genomic DNA was extracted from frozen tissue samples using the DNeasy Blood and Tissue kit (QIAGEN Ltd, UK) as per manufacturer's instructions. DNA was eluted with purified (deionized double-distilled) H_2_O and stored at -20°C until quantification. DNA purity and concentration was ascertained by use of a Nanodrop™ 1000 spectrophotometer. Samples had a 260/280 nm absorbance ratio between 1.8-2.0 and were diluted in purified H_2_O to ~5 ng/μl prior to PCR.

#### Cervical intra-epithelial neoplasia (CIN) or vulval intra-epithelial neoplasia (VIN) samples

Total genomic DNA was extracted as described previously [26] and stored at -20°C until quantification. Standard strict precautions for prevention of contamination and false positives (for DNA extraction and all PCR procedures) were observed [27].

#### Oropharyngeal cancer samples

Oropharyngeal samples were disrupted in a Bullet Blender™ (Next Advance, Averill Park, USA) in 300 μl digestion mix (10 mM Tris, pH 7.5; 10 mM EDTA; 0.5% SDS; 200 μg/ml Proteinase K) for 5 minutes and then incubated o/n at 37°C. Following Proteinase K inactivation at 56°C for 10 minutes, the lysate was subjected to a phenol:choloroform extraction (1:1 volume) and the supernatant precipitated with 1 ml 100% ethanol. The DNA was then centrifuged (13,000 rpm, 4°C, 20 mins), the pellet washed with 70% ethanol, air dried and resuspended in 200 μl PBS. RNase digestion and total genomic DNA isolation was then performed using the DNeasy Blood and Tissue Kit (QIAGEN Ltd, UK), according to the manufacturer's instructions and eluted quantified and stored as outlined above.

### PCR methods

#### Single step PCR analysis

A PCR assay using the PGMY09/11 L1 consensus primer set was performed as described previously [24]. A 1.5% agarose gel (in TBE) was then used to confirm the presence/absence of bands specific for both HPV and human β-globin. Similarly, PCRs using the GP5+/6+ and MY09/11 primers were performed as previously described [19,21]. The PCR cycling conditions were as follows; PGMY09/11 and MY09/11 primer sets: denaturing step of 95°C for 5 min, followed by 40 cycles of 95°C for 1 min, 55°C for 1 min and 72°C for 1 min. This was followed by a final extension period of 10 min at 72°C. GP5+/GP6+ primer set: denaturing step of 95°C for 5 min, followed by 40 cycles of 95°C for 1 min, 40°C for 2 min and 72°C for 1.5 min. This was followed by a final extension period of 10 min at 72°C. The sequences of the primers used are shown in Table 1.

**Table 1:** Primers used to detect HPV in clinical samples.

Primer Set	Primer name	5'-3' sequence
GP5+/GP6+	GP5+	TTT GTT ACT GTG GTA GAT ACT AC
	
	GP6+	GAA AAA TAA ACT GTA AAT CAT ATT C

MY09/11	MY09	CGT CCM ARR GGA WAC TGA TC
	
	MY11	GCM CAG GGW CAT AAY AAT GG

PGMY09/11	PGMY11-A	GCA CAG GGA CAT AAC AAT GG
	
	PGMY11-B	GCG CAG GGC CAC AAT AAT GG
	
	PGMY11-C	GCA CAG GGA CAT AAT AAT GG
	
	PGMY11-D	GCC CAG GGC CAC AAC AAT GG
	
	PGMY11-E	GCT CAG GGT TTA AAC AAT GG
	
	PGMY09-F	CGT CCC AAA GGA AAC TGA TC
	
	PGMY09-G	CGA CCT AAA GGA AAC TGA TC
	
	PGMY09-H	CGT CCA AAA GGA AAC TGA TC
	
	PGMY09-I	G CCA AGG GGA AAC TGA TC
	
	PGMY09-J	CGT CCC AAA GGA TAC TGA TC
	
	PGMY09-K	CGT CCA AGG GGA TAC TGA TC
	
	PGMY09-L	CGA CCT AAA GGG AAT TGA TC
	
	PGMY09-M	CGA CCT AGT GGA AAT TGA TC
	
	PGMY09-N	CGA CCA AGG GGA TAT TGA TC
	
	PGMY09-P	G CCC AAC GGA AAC TGA TC
	
	PGMY09-Q	CGA CCC AAG GGA AAC TGG TC
	
	PGMY09-R	CGT CCT AAA GGA AAC TGG TC
	
	HMB01	GCG ACC CAA TGC AAA TTG GT
	
	GH2O	GAA GAG CCA AGG ACA GGT AC
	
	PCO4	CAA CTT CAT CCA CGT TCA CC

Primer sets, names and sequences used for the detection of HPV in clinical samples. GP5+/GP6+ and MY09/11 PCRs consist of a single forward and reverse primers, whereas the PGMY09/11 set comprises 5 forward (11A-E) and 13 reverse (09F-HMB01) primers and includes GH2O and PCO4 β-actin internal controls.

#### Nested PCR and direct cycle sequencing

PCR reactions that were negative following amplification with the PGMY09/11 L1 consensus primers were subjected to a further 30 rounds of PCR amplification using the GP5+/GP6+ primer pair as described previously [19].

Positive bands on a gel were excised, the DNA purified using QiaQuick Gel Extraction columns (QIAGEN Ltd, UK) and sequenced directly (Geneservice Ltd, UK). The sequences were then aligned with known HPV types (NCBI Basic Local Alignment Search Tool).

#### Sensitivity of the single and nested PCR approaches

Amplification of serial dilutions of HPV6, HPV16 and HPV18 plasmids demonstrated equal sensitivity of the MY, GP and PGMY primer sets at 1-10 copies per cell input (data not shown). We found that the PGMY/GP nested PCR system was able to perform consistently at a high level of sensitivity, namely 0.1-1 copy per cell input (data not shown). This conforms to the requirements of the World Health Organisation for the proficient detection of HPV DNA [28].

#### PGMY-line blot assay/Linear Array HPV genotyping test (LA HPV GT) (Roche Diagnostics Ltd., UK)

The procedure was carried out according to the manufacturer's instructions and as previously described [26]. Briefly, PCR amplification was carried out with LA HPV GT primers as provided: Each 100 μl reaction consisted of 50 μl working master mix containing MgCl2, KCl, Amplitaq Gold DNA polymerase, uracil-N-glycosilase, deoxynucleotides (dNTPs) and biotinylated PGMY and β-globin primers together with 50 μl of DNA sample. DNA templates were titrated to a concentration of 2-4 ng/μl, i.e. 100-200 ng template DNA per reaction. The Applied Biosystems Gold-plated 96-Well GeneAmp PCR System 9700 was programmed as follows: 50°C for 2 min, 95°C for 9 min and 40 cycles of 95°C for 30 s, 55°C for 1 min, 72°C for 1 min and finally, at 72°C for 5 min before holding it indefinitely at 72°C. The 40 cycles had a ramp rate set at 50%.

Hybridization to the oligonucleotide probe: 100 μl of denaturing solution (DS) was added to the PCR product. All washes and hybridization steps were undertaken in a 24-well tray with lid. The denatured amplicons were hybridized on to the strip containing specific probes for 37 HPV genotypes and β-globin reference lines before undergoing stringent washes.

Colorimetric determination with a Linear Array Detection Kit: the colour change reaction was from streptavidin-horseradish peroxidase mediated precipitation of working substrate. Positive reactions appeared as blue lines on the strip. The strips were interpreted using the HPV reference guide provided.

### Statistical methods

The unweighted Kappa statistic was calculated to assess the inter-assay concordance and agreement for the rates of HPV positivity in the clinical samples. Kappa values of 0-0.2 (slight), 0.21-0.4 (fair), 0.41-0.6 (moderate), 0.61-0.8 (substantial) and 0.81-1.0 (almost perfect) indicated the level of agreement between the methods used [29]. Statistical analyses were performed with SPSS software.

## Results

### Head to head PCR amplification results on warts, cervical brushings and vulval specimens

MY and GP primers detected HPV in 9/13 wart samples (69.2%), whilst PGMY primers detected HPV in all warts (Table 2). For the cervical brush samples, MY primers detected HPV in 9/10 samples (90.0%), whilst GP primers detected HPV in 8/10 samples (80.0%). Again, PGMY primers detected HPV in all CIN samples. All three primer sets had the lowest detection rates for vulval samples; HPV presence was determined in 7/11 (63.6%), 6/11 (54.5%) and 6/11 (54.5%) VINs for MY, GP and PGMY primers sets, respectively. We therefore performed a nested PCR with the PGMY amplicons using the GP5+/GP6+ primers, on all samples demonstrating a negative or inconclusive PGMY result. Statistical analysis indicated a moderate agreement when comparing the three PCR methods with each other in all samples; MY and GP κ = 0.436 (agreement 76.5%), MY and PGMY κ = 0.472 (agreement 82.4%), GP and PGMY κ = 0.530 (agreement 82.4%). The overall agreement between the three methods for all samples was 70.6% (24/34).

**Table 2:** PCR results with 3 primer sets on different clinical samples.

Patient	Sample	MY	GP5+/GP6+	PGMY
1	W1	-	+	+

2	W2	+	-	+

3	W3	+	-	+

4	W4	+	+	+

5	W5	+	+	+

6	W6	-	+	+

7	W7	+	+	+

8	W8	-	+	+

9	W9	+	-	+

10	W10	-	-	+

11	W11	+	+	+

12	W12	+	+	+

13	W13	+	+	+

14	C1	+	+	+

15	C2	+	+	+

16	C3	+	+	+

17	C4	+	-	+

18	C5	-	-	+

19	C6	+	+	+

20	C7	+	+	+

21	C8	+	+	+

22	C9	+	+	+

23	C10	+	+	+

24	V1	+	+	+

25	V2_pre_	+	+	+
	
	V2_post_	-	-	-

26	V3_pre_	+	+	+
	
	V3_on_	+	+	+
	
	V3_post_	+	+	+

27	V4_on_	+	-	-
	
	V4_post_	-	-	-

28	V5_pre_	+	+	+

29	V6_on_	-	-	-
	
	V6_post_	-	-	-

Wart (W), CIN (C) and VIN (V) samples were tested for HPV using three different primers sets. The PGMY primers detected HPV in more wart and CIN samples than either MY or GP5+/GP6+ PCR systems. There were no significant differences between the three sets when VIN samples were analysed. VIN samples collected pre-, on- or post- Imiquimod treatment are indicated.

Nested PGMY/GP PCR detected HPV in all but one sample (sample V2_post_), a biopsy obtained after Imiquimod treatment for VIN 2 disease. We sequenced the nested amplicons to confirm true HPV genotype amplification, and compared these results with those obtained by HPV Linear Array (Roche Diagnostics Ltd, UK) (Table 3). The results were consistent, but sequencing revealed a subtype not detected by the Linear Array from sample C5, namely HPV87. The Linear Array also detected an additional subtype compared to direct sequencing in two samples, HPV52m in C4 and HPV54 in V4_post_. Linear array of sample V2_post _revealed HPV subtypes 45 and 54, which remained undetected by nested PCR. Sample V4_on _was the only sample where a typing discrepancy was evident.

**Table 3:** Nested PCR with the PGMY-GP system on previously negative or inconclusive results after primary PCR screen.

Sample	PGMY-GP nested PCR	HPV sequenced	Linear Array
C4	+	58	52m, 58

C5	+	87	-

V2_post_	-	ND	45, 54

V4_on_	+	16	54

V4_post_	+	16	16, 54

V6_on_	+	81	81

V6_post_	+	16	16

Samples negative or weakly positive for HPV using any of the three PCR primer sets were re-amplified using a PGMY-GP5+/GP6+ nested approach. Positive samples were directly sequenced to determine HPV type and the result compared to Linear Array.

### Direct comparison of primary PGMY screening, Linear Array™ and nested PGMY-GP with sequencing on oropharyngeal cancers

PGMY PCR screening of oropharyngeal cancer specimens showed that no samples were initially positive, although 3 samples proved positive when tested with Linear Array™. Furthermore, 10 specimens proved positive for HPV when tested with the nested PCR system (PGMY-GP), as confirmed by direct sequencing of the amplified DNA (Table 4).

**Table 4:** HPV detection and typing of 15 HNSCC and 3 RRP patients.

			HPV detection method		
			
Patient	Age	Diagnosis	PGMY 09/11	Linear Array	GP5+/GP6+ nest
1	52	SCC	-	-	HPV 6

2	46	SCC	-	-	HPV 6

3	49	SCC	-	-	HPV 16, mixed

4	58	RRP	-	-	-

5	68	SCC	-	HPV 16	HPV 16

6	31	RRP	HPV 11	HPV 11	HPV 11

7	61	SCC	-	-	-

8	68	SCC	-	-	-

9	54	SCC	-	HPV 16	HPV 6

10	64	SCC	-	-	HPV 6

11	72	SCC	-	-	HPV 16

12	73	SCC	-	HPV 11	HPV 6

13	87	RRP	-	-	-

14	69	SCC	-	-	-

15	74	SCC	-	-	-

16	50	SCC	-	-	HPV 40

17	78	SCC	-	-	mixed types

18	74	SCC	-	-	-

HPV +ve HNSCC detection			0/15	3/15	10/15	

PCR detection, using the PGMY09/11 primer set was unable to detect the presence of HPV in any of the head and neck squamous cell carcinoma (SCC) samples (0/15). Improved detection was achieved using the Linear Array assay (3/15) and following a nested PCR of the PGMY reaction with the GP5+/GP6+ primer set (10/15). HPV was only detected in 1/3 of recurrent respiratory papillomatosis (RRP) samples, irrespective of the method used. Samples were scored as positive if HPV was detected using one or more of the methods employed.

## Discussion

There is a need for better and more time-, labour- and cost-efficient detection of HPV from clinical samples. For many years, the focus has been on detection of HPV from cervical samples, and the PCR systems most frequently used worldwide have used the degenerate/consensus primers MY09/11 and GP5+/6+ [19-22]. More recently, the non-degenerate PGMY09/11 primers have been developed, and shown to detect a broader range of subtypes from cervical samples with better sensitivity [24].

Earlier epidemiological studies predominantly used the MY and GP primer sets to detect HPV infection in cervical cancers, but it was only with increased sensitivity assays such as nested PCRs that cervical cancers worldwide were found to be 99.7% HPV positive [3]. Munoz et al. used both sets of degenerate primers in a multi-centre case-control study detailing the risks of cervical cancer associated with different HPV subtypes and found a HPV detection rate of 90.7% [5]. We tested three primer sets on 34 blinded samples from 30 patients, including samples from warts, cervical brushings and vulval biopsies to give a range of clinical tissue samples. Genital warts and cervical brushings from CIN lesions are essentially 100% positive for HPV ano-genital subtypes and were used as positive controls. We then directly compared PGMY primary screening, nested PCR (PGMY-GP), and the Linear Array™ system on DNA extracted from oropharyngeal cancers.

The PGMY primers performed very well compared to the MY and GP primers, in the detection of HPV from genital warts. Identification of HPV subtypes in warts is not routinely undertaken, as the vast majority of warts have been shown to be caused by just two subtypes, HPV 6 and 11 [30,31]. However, clinically detectable warts may mask the presence of HR-HPV infection, particularly in immunosuppressed individuals such as transplant patients or HIV+ patients. These patients commonly harbour multiple HPV subtype infections and may have increased predisposition to malignancy with HR-HPV. For example, HIV+ men who have sex with men (MSM) have been found to have a vastly increased rate of anal cancer [32]. Therefore, there is a requirement for increased screening and identification of HPV at non-cervical sites.

HPV detection rates in vulval biopsy samples were low for all three primer sets, probably due to low copy number infection in the samples, or the presence of subtypes not detected by these primer sets. Also, many samples were obtained during treatment of patients, decreasing HPV viral load (per cell) as patients respond (rev. [33]). In order to reduce the possibility of false negative results, we therefore employed an ultra-sensitive nested PCR (PGMY-GP5+/6+) according to the protocol established by Fuessel Haws et al. [25]. This confirmed that the vast majority of the negative results obtained following primary PCR were due to insufficient sensitivity. However, Fuessel Haws et al. also reported a significant problem with false positives, whereby a sequence from human genomic DNA was commonly amplified. We felt it was important to sequence the amplicons for confirmation that HPV was truly present in the sample. All but one sample (V2_post_) were HPV positive, thereby these data show that PGMY PCR may be useful as first-line PCR screening, but is not sufficiently sensitive to detect HPV in vulval or head and neck cancer samples. We suggest that nested PCR should be performed on clinical specimens if the initial PGMY PCR is negative, if only to confirm HPV negativity.

It is interesting to note that despite treatment and clinical resolution of the vulval lesions, we were able to detect the presence of HPV in all of the samples tested. The same HPV subtype was present in the post-treatment sample V4_post_. The continued presence of a HR-HPV subtype in the lesion means that the clinician and patient may need to continue monitoring of the site for clinical recurrence, suggesting the use of PCR detection of HPV to inform clinicians of the need for continued surveillance of the infected site(s).

**Figure 1:**
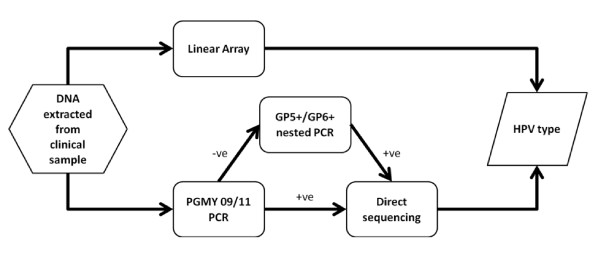
**Methodological flowchart of DNA analyses performed**. DNA extracted from clinical samples was subjected to both Linear Array and PGMY09/11 PCR analysis. In the event of a positive Linear Array result, the HPV subtype was known immediately. In the case of a positive PGMY result, direct sequencing (using the GP5+ internal primer) enabled the subtype present to be determined. In the case of a PGMY negative result, a further nested PCR amplification step was performed using the GP5+/GP6+ primer set. Subsequent positive results were then directly sequenced using the GP5+ primer alone. Samples negative for both Linear Array and nested PCR were classified as free from infection.

When we applied our regime to oropharyngeal cancers, we found that the nested PCR system was the most sensitive method. The results are consistent with previous work showing that determination of HPV DNA viral loads from the oral cavity were better performed with a nested PCR system [34], though contrast with recent published work detecting HPV in oral rinses or swabs with the Linear Array™ system, type-specific PCR primers or consensus primers [35-39]. Interestingly, the only HPV positive RRP was taken from a 31 year old patient, the only sample to be taken within the usual age range for adult onset of the disease [40]. The mean ages of patients with HPV positive HNSCC was 60.6 years, compared to 69.2 years in patients with HPV-unrelated disease (n = 15, p = 0.1645, Mann Whitney U-test). This is consistent with data from a large US study of HPV prevalence in oral SCCs [41], indicating earlier onset of HPV-related malignancies of the oral cavity and highlighting pathological differences between HPV-related and HPV-unrelated disease. The Linear Array™, incorporating an amplification step, was more sensitive (determined sensitivity for each subtype is provided in the manufacturer's manual) than a pure PGMY primary screening. Our work suggests that even with direct testing of tumour material, the Linear Array™ system or "normal" PCR with consensus primers may not be sufficiently sensitive to detect HPV DNA present at low copy number. This may be important as HPV may be present as a single copy per cell in advanced pre-malignant or malignant lesions, associated with high-risk HPV integration. Figure 1 illustrates a suggested protocol for the detection of HPV in a range of clinical samples.

The performance and type-specific sensitivity of the MY, GP and PGMY PCR systems have been previously compared and reviewed. The GP and MY primer sets amplify a wide range of HPV types, with the MY system being shown to detect significantly more infections with multiple types [42]. Type-specific amplification differences are also evident between the two systems, probably reflecting the degenerate primers of the MY PCR and the consensus primers of the GP PCR. The PGMY system has been demonstrated to have a type-specific increase in amplification efficiency, when compared to the MY system from which it is derived [24]. These findings were confirmed in this study, PGMY detecting HPV in the greatest number of clinical samples (29/34), followed by MY (25/34) and GP (23/34) primer sets. The use of a nested PCR, using GP5+/GP6+ primers to further amplify PCR products generated by the PGMY09/11 primer set, has been demonstrated to be both highly sensitive and capable of detecting more HPV types per sample [25]. Our use of this method to detect HPV in oropharyngeal tumours improved detection from 0/15 to 10/15 samples, compared to a single round of PGMY PCR, demonstrating its potential value in the analysis of HNSCC samples.

A PCR approach to HPV detection in clinical samples is less expensive than the use of the Linear Array™ system. However, the latter enables detection of multiple HPVs (37 distinct types) whereas sequencing following PCR detection often reveals only a single subtype [23], dependent on both viral DNA load and primer binding affinity. Given that HPV-related HNSCC is clinically different from HPV-unrelated HNSCC, with improved survival and lower rates of disease recurrence [43], it is anticipated that the sensitive detection of HPV DNA in such lesions will inform both prognosis and treatment.

## Conclusions

Our results suggest that the older and commonly used MY09/11 and GP5+/GP6+ primer sets may not be sufficient for primary HPV detection from non-cervical clinical samples, and that negative results with primary PGMY PCR screening should be considered for a GP5+/GP6+ nested PCR. However, the detection of HPV alone from clinical samples does not automatically lead to the conclusion that HPV is involved in the causation of the lesion. Detection of HPV may be due to HPV "bystanders" or contamination of the sample, due to the ubiquity of HPV on skin and mucosal surfaces in the human population. Evidence that HPV is involved in the pathogenesis of clinical disease requires the demonstration of transcriptionally active virus in lesional cells. In particular, the demonstration of E6 and E7 oncogenic activity is the "gold standard" by which HPV activity is measured, therefore future HPV detection methods should be supplemented by E6/E7 detection, for example using quantitative real-time PCR.

## Abbreviations

HPV: human papillomavirus; PCR: polymerase chain reaction; MSM: men who have sex with men; HIV: human immunodeficiency virus; TBE: Tris/Borate/EDTA; HR: high risk; LR: low risk; HG: high grade; LG: low grade.

## Competing interests

This work was supported by grants from the British Skin Foundation and Cancer Research UK to PKCG. PKCG and MAS act as consultants to Sanofi Pasteur-MSD, Lyon, France and are in receipt of an unrestricted educational grant. MAS also acts as consultant to Merck Research Laboratories, Westpoint, USA, and GSK Biologicals, Rixensart, Belgium.

## Authors' contributions

DMW carried out a majority of the experiments, analysed data and helped write and draft the manuscript. SLRB carried out experiments, analysed data and helped plan and draft the manuscript. KV carried out experiments and helped draft the manuscript. NH helped plan the study, redrafted the manuscript and provided clinical samples. YLW helped draft the manuscript, analysed data, carried out experiments and provided clinical samples. J-TF provided clinical samples and helped draft the manuscript. JCS helped plan the study, redrafted the manuscript and provided clinical samples. MAS helped plan the study and redrafted the manuscript. HS helped plan the study, redrafted the manuscript and provided clinical samples. PKCG conceived and planned the study, participated in its design and coordination, provided clinical samples and wrote the manuscript. All authors read and approved the final manuscript.

## Pre-publication history

The pre-publication history for this paper can be accessed here:

http://www.biomedcentral.com/1471-2407/9/440/prepub
